# Qualitative and Artificial Intelligence-Based Sentiment Analysis of Turkish Twitter Messages Related to Autism Spectrum Disorders

**DOI:** 10.7759/cureus.38446

**Published:** 2023-05-02

**Authors:** Pelin Göksel, Volkan Oban, Gül Dikeç, Miraç Barış Usta

**Affiliations:** 1 Psychiatry, Ondokuz Mayıs University, Samsun, TUR; 2 Psychiatry, Independent Reseacher, Istanbul, TUR; 3 Nursing, Fenerbahce University, Istanbul, TUR; 4 Child Psychiatry, Ondokuz Mayıs University, Samsun, TUR

**Keywords:** health public, emotion analysis, twitter messagging, social media, autism spectrum disorder (asd)

## Abstract

Background: The aim of our study was to conduct an emotional analysis of Turkish Twitter messages related to autism spectrum disorders (ASD).

Methods: An emotion analysis was performed using quantitative and qualitative analysis methods on Turkish Twitter messages shared between November 2021 and January 2022 that contained the words "autism" and "autistic."

Results: It was found that 81.5% of the 13,042 messages that constituted the sample of this study contained neutral emotions. The most frequently used words in Twitter messages were autism, a, universe, strong, patience, warriors, and happy. The qualitative analysis revealed three main themes. These themes were: "experiences," "informing society and awareness," and "humiliation."

Conclusion: In this study, it was found that Turkish Twitter messages related to autism, which were analyzed using artificial intelligence-based emotion analysis, often contained neutral emotions. While the content of these messages, often shared by parents, was related to experiences, and the messages shared by pediatric psychiatrists and rehabilitation center employees were informative in nature, it was determined that the word "autism" was used to insult, which is outside of its medical meaning.

## Introduction

Autism Spectrum Disorder (ASD) is a congenital neurodevelopmental disorder with symptoms first observed in early childhood. Autism, which is characterized by limited interests and repetitive behavior patterns as well as significant differences in social communication and interaction, manifests itself in the early stages of development and results in issues in social functionality areas [1]. A child with autism causes severe anxiety in the family for many reasons, including the uncertainty of the diagnosis, the severity and duration of the disorder, and the child's non-compliant behavior with social rules. On the other hand, the level of knowledge about autism in society is very low, and it is often equated with mental retardation and seen as a disease that can't be treated [2].

Stigmatization is the act of other people degrading the dignity of people living in a society for not fitting the standards that society considers "normal" [1]. The fact that children's behaviors do not comply with the definitions of normality in autism causes children to be labeled and stigmatized as different and/or strange, which also affects the child's family. In other words, these children are excluded for not having the qualities or not being able to fulfill the behavior patterns that are expected of them [2]. Therefore family members of these individuals may avoid social environments, consume their time and energy to hide the disorder, be discriminated against in social areas, or stay at home at all times [3]. Both the prevalence of autism and the fact that it affects both the person with autism and the people around them make autism and people's approach to it a very important social problem [2].

Social media are social technology tools that are based on the idea of sharing and that allow individuals to build networks or actively participate in existing networks [4]. According to the Pew Research Center (2021), the most popular social media platforms were determined to be Youtube, Facebook, Instagram, Pinterest, LinkedIn, Snapchat, Twitter, and WhatsApp, respectively. Most of the users visit these platforms daily [5]. In this study, Twitter data were selected to measure the stigmatization of autism and autism-related words based on the ease of access and popularity criteria of the data, and the data will be analyzed using artificial intelligence methods.

In alignment with the development and spread of artificial intelligence techniques, emotion analysis is an analytic method that is frequently used in analyzing data from online applications, particularly in recent years, and that helps determine the emotions underlying texts and positive or negative conditions in these texts. It allows for the determination of people's opinions, attitudes, and emotions [6]. As one of the techniques for natural language processing, emotion analysis is the most frequently used method for this purpose and is addressed with two approaches [7]. In the international literature, there are very limited studies dealing with autism-related messages. When the Turkish literature was examined, no study on the topic was found. This could be due to difficulties encountered during the Turkish analysis of messages. Accordingly, the aim of this study is to examine Turkish messages in terms of stigmatization by using the term "autism," which is a disorder frequently seen in childhood and causes stigma. This study contributes to the literature by using artificial intelligence applications, performing emotion analysis, and performing qualitative analysis by researchers.

The aim of this study was to perform an emotional analysis of Turkish Twitter messages using the term "autism."

## Materials and methods

Study design

This is a descriptive study conducted with quantitative and qualitative methods on Twitter messages, and the data were collected retrospectively. As the study was designed as descriptive, there are no research hypotheses, and the research question guiding the study is: What are the results of sentiment analysis of Twitter messages about autism written in Turkish?

Research setting and time

The study's data were collected in July 2022 using the Python Library Tweepy application, which is an application used to access the Twitter database, which provides to get live and real-time tweets and to perform sentiment analysis [8].

Research sample

All Turkish Twitter messages shared between November 2021 and January 2022 constituted the population of the study, and 13042 Turkish Twitter messages that included the "autism" and "autistic" keywords constituted the sample. Messages that included the words "autism" and "autistic" were used in the emotion analysis, which is the first phase of the study. In the second phase of the study, messages that were most frequently liked, reshared, and reached more people-which constituted approximately 23% of the total number of messages-were evaluated within the scope of qualitative data analysis. The criterion sampling method, which is one of the purposive sampling methods and used for situations that meet a set of predetermined criteria, was used in qualitative data analysis [9]. There is no accepted sample size formula recommended for use in qualitative research [10]. The most frequently used method in determining the sample size in purposive sampling methods is to stop data collection at the point where no different information comes from the new sample units, that is, at the saturation point [11]. In our study, based on the criteria described, criterion sampling method was used in accordance with the criteria we determined before (scanning tweets containing autism and autism keywords), and data collection was terminated when the data set reached saturation.

Data analysis

In the study, The Strengthening the Reporting of Observational Studies in Epidemiology (STROBE) guidelines were used in reporting the quantitative data of the study [12], while Consolidated Criteria for Reporting Qualitative Studies (COREQ) guidelines were used in reporting the qualitative data [13].

In the quantitative and emotional analysis of the data, "Bidirectional Encoder Representations from Transformers (BERT)", one of the natural language processing (NLP) methods, was used for feature extraction from messages. BERT is a state-of-the-art method, a technique that Google uses in 70 languages as of October 2019 and is used in almost all search engines for English. In this study, a pre-trained feature extraction technique for Turkish called BERTurk was used [14]. The classification was done with the Multilayer Perceptron (MLP), one of the Artificial Neural Networks (ANN) that is frequently used in psychiatry and behavioral sciences [7].

The qualitative data analysis in the second phase of the study was completed by the two researchers who took part in the study. In the qualitative phase, 3000 messages that were the most frequently liked and re-shared, for which emotion analysis was performed, were analyzed using Colaizzi's phenomenological interpretation method [15]. The steps (1-6) of Collaizi's phenomenological data analysis were completed as follows: 1. The messages were read and re-read to understand the emotions in the messages. 2. Significant statements related to autism were selected. 3. These significant statements were examined to formulate meanings. 4. The formulated meanings were grouped into themes and sub-themes. 5. The results obtained were combined in a rich and comprehensive manner. 6. The fundamental conceptual structure of the messages were defined. Moreover, words that are used most frequently in the messages were read with Tweet Text, and a word cloud was created with the words, which are visualized as larger when they are used frequently and smaller when they are used infrequently.

Ethical aspects of the study

The ethics committee approval of this study was obtained from the X University Non-Interventional Clinical Research Ethics Committee, with the approval date 08.06.2022 and number E-67888467-204.01.07-8822. Individual informed consent was not obtained as the study was conducted on social media, which is a public platform, institutional approval had been obtained, and the data were anonymous. When extracting data from Twitter, the Personal Data Protection Law (2016) and the Internet Research Ethics Guide [16] were followed, and the personal information of the individuals was not collected. Institutions, organizations, hashtags (#), mentions (@), and website links (URL addresses) were removed from the messages. The selection of the messages to be included in the qualitative analysis was performed by another researcher who did not do the qualitative analysis in the study. The messages were ranked as the most frequently liked and re-shared. Then, the column containing usernames was deleted. The names, institutions, and organizations mentioned in the messages in the text were coded as X, Y, Z, and A, B, and the anonymity of individuals was ensured.

## Results

Quantitative results

**Figure 1:**
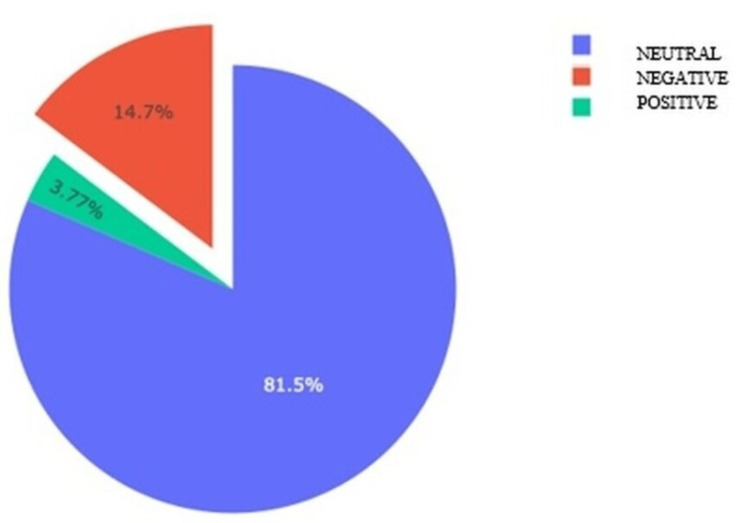
Emotion Analysis of Messages Related to Autism

**Figure 2:**
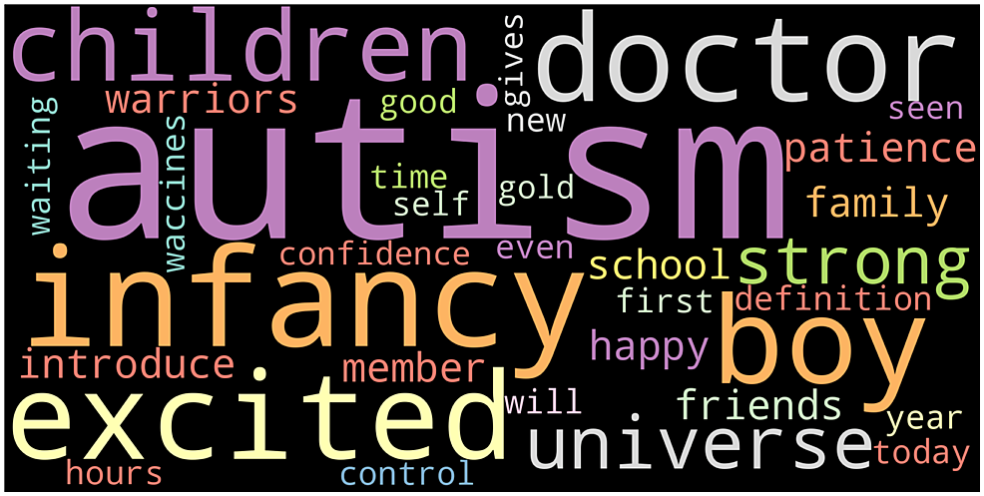
Word Cloud Created by Text-to-Speech from Autism-Related Messages

It was determined that 81.5% of the 13,042 messages analyzed within the scope of the study contained neutral emotions (Figure 1). A word cloud was prepared with the most frequently used words in the messages using the text-to-speech method, which is shown in Figure 2. 

Qualitative findings

Three themes were identified as a result of the qualitative analysis. These themes were experiences, informing society and awareness, and humiliation.

Experiences

It was determined that this theme mostly consisted of the experiences of parents and teachers of children with an autism spectrum disorder. The parents of children diagnosed with autism shared their diagnosis processes, their experiences in daily life, and when they have challenges in their messages. 

"X wished a happy week to all friends when s/he was on the way to school. Have a good day."

“The most powerful warriors in the universe are patience and time. We have the determination that will not go away with time but will give us more strength over time.”

"He and his father went fishing after school. My son X takes everything he does seriously."

“I want you to hear me; lend an ear to my cry now. It is difficult to take care of a child with autism on my own. I'm not complaining, but parents of children with autism would understand this.”

“I went for a walk for an hour today, and when I got home, Y was crying. S/he cried for me until I came home.”

Informing society and awareness

It was determined in this theme that child and adolescent psychiatrists frequently share messages to inform society. It was also found that special education and rehabilitation centers, as well as municipalities, provide information about the practices in the centers.

"Autism is not a disease, but a difference. As it is not a disease, there is no treatment, but there is personalized education to increase their adaptation to society."

“Only a child psychiatrist can diagnose autism; only a special education specialist can deliver special education; only an occupational therapist can do occupational therapy.”

“In our Autism Support Center, we use an internationally valid education model and increase the quality of education for our children with autism. We will continue to work with the Tohum Autism Foundation and will always be there for children with autism.”

“Autism, which occurs in 1 in 54 children, is a neurodevelopmental difference that occurs congenitally or in the first years of life. Be aware. Create awareness."

Humiliation

In some of the messages examined, it was determined that the word "autism" was used for insult and humiliation purposes other than diagnosis. In the messages, it was determined that individuals were labeled autistic, particularly because they could not establish a relationship.

“Autistic stupid idiot f... the team you go to….”

“My autistic brother can't communicate with girls like a prick who has lived in the monastery from middle school to high school”

“Autistic b.., you ruined the woman.”

“My loved ones have become autistic towards me…”

“You shouldn't have gone to Paris; you would get a virus, you would be.., you autistic…”

## Discussion

The aim of our study was to conduct emotional analysis of Turkish Twitter messages related to autism using quantitative and qualitative methods. The qualitative analysis revealed three major themes: experiences, informing society and awareness, and humiliation.

The main result we reached with quantitative data analysis is that 81.5% of Turkish Twitter messages contained neutral emotions, and 14.7% had negative emotions. A study focusing on the social media analysis of Twitter messages related to autism between 2019 and 2020 categorized the messages by topics and showed that the messages containing neutral emotions were mostly related to spreading information, the messages containing positive emotions were mostly related to the necessity of providing consistent support and the messages with negative emotions were mostly related to criticism directed at the system. Considering all the messages in the study, the data analysis did not focus on identifying the emotion that was frequently used in the messages; however, a topic-based evaluation revealed that there are fewer messages containing neutral emotions in general [17]. In the study conducted by Bellon-Harn et al. [18], the number of Twitter messages containing positive emotions about autism was found to be significantly higher than those containing negative emotions (Mean of positive emotions/negative emotions: 4.2/1.8). More studies are needed to make comparisons on the subject.

The word cloud created showed that the words that were used more frequently were autism, one, universe, strong, patience, warriors, and happy. This finding indicates that many Twitter messages are shared for support and motivation purposes. An examination of the literature showed that there are studies demonstrating that messages containing positive feelings about autism and intended to provide support are frequently shared, consistent with the findings of our study [17,18].

The main theme of "experience" generally consisted of messages shared by parents. Some parents mentioned their children's positive character traits and behaviors, including seriousness, kindness, and hard work. Similar to our findings, Cost et al. conducted a study with the parents of children with autism using qualitative methods and found that parents mostly saw positive character traits in their children and that happiness, kindness, and being loving were the most frequently described traits [19].

Some parents discussed the challenging nature of taking care of a child with autism and the lack of resources for support. There are many studies in the literature reporting similar results [20,21]. A recent study conducted with caregivers of a child with autism showed that families with children with autism had lower leisure time and family life satisfaction compared to typical families [22]. According to a qualitative study conducted by Bravo-Benitez et al., reasons such as the uncertainty about the course of symptoms and the inability of children to reach the developmental stages revealed feelings of grief in caregivers, and support programs for caregivers were needed [23].

Informative and awareness-raising messages related to the symptoms of autism and appropriate interventions stood out in the main theme of informing society and awareness. When the literature is reviewed on the subject, studies concluding that both parents and healthcare professionals do not have sufficient knowledge about autism are encountered [24,25]. A study evaluating the outcomes of an education program designed for autism symptoms showed that after the program, service providers referred more children with autism suspicion, suggesting that there is a need for policies toward autism findings and early interventions that can be applied to the whole population [26].

The humiliation theme consisted of the messages in which the word autism is used to insult. It was found that the users who write these messages generally focus on the social communication difficulties experienced by the people they insult. Research on the use of the word "autism" in the media reports conflicting conclusions. Prochnow A [27] started with the hypothesis that the word "autistic" is often used in the media with an insulting meaning, but the study results did not support the hypothesis; on the contrary, they found many overly positive comments, attributing this to the fear of political misunderstanding in the media. It is particularly noteworthy that the word autism has recently been used on social media platforms to refer to people who practice marginalized practices for the public's benefit and justice and who are technologically skilled but have weak social skills. Online hate speech is on the rise, reaching an alarming level [28].

The fact that the sentiment analysis cannot be done perfectly and the irony cannot be detected creates a limitation. Considering only Turkish Twitter messages is also a limitation. In addition, the social, economic and political environment in which users and communities live can affect the content and, therefore, the sensitivity of their tweets. This situation limits sentiment analysis.

## Conclusions

In our study, we concluded that social media focuses on experiences, awareness, and information sharing in autism-related messages. Apart from this, another result was that the word autism was used as an insult by some users. Future studies on the subject can be repeated on different social media platforms or sentiment analysis of the created themes can be done within themselves. It can be suggested that studies using emotion analysis in the age of artificial intelligence, where individuals can share their feelings and thoughts on almost every subject uncensored through social media, should be carried out not only for autism but also for many mental illnesses with high stigmatization. The number of anti-stigma campaigns that can be carried out in online environments can be increased and their effects measured. In addition, active use of social media by patients diagnosed with mental disorders and their relatives can be an important way to combat stigma.
